# Supplementary material for: The Effects of Creatine Supplementation on Upper- and Lower-Body Strength and Power: A Systematic Review and Meta-Analysis
Source: Nutrients. 2025 Aug 25;17(17):2748. doi: 10.3390/nu17172748 (PMC12430374; doi:10.3390/nu17172748)
Supplement: Supplementary file 1 [file nutrients-17-02748-s001.zip › nutrients-3776589-supplementary.pdf]

Supplemental Table S1. Search strategy.

Databases	Search strategy	Limits	Results
PubMed	("creatine"[Title/Abstract] OR "creatine supplementation"[Title/Abstract]) AND ("performance"[Title/Abstract] OR "strength"[Title/Abstract] OR "muscle strength"[Title/Abstract] OR "exercise performance"[Title/Abstract] OR "athletic performance"[Title/Abstract] OR "sports performance"[Title/Abstract] OR "force"[Title/Abstract] OR "muscular strength"[Title/Abstract] OR "muscular force"[Title/Abstract])	Article, English	3399
Scopus	("creatine" or "creatine supplementation") AND ("performance" or "strength" or "muscle strength" or "exercise performance" or "athletic performance" or "sports performance" or "force" or "muscular strength" or "muscular force")	Article, English	11585
Web of science	("creatine" or "creatine supplementation") AND ("performance" or "strength" or "muscle strength" or "exercise performance" or "athletic performance" or "sports performance" or "force" or "muscular strength" or "muscular force")	Article, English	7574

Supplementary Table S2. Quality assessment.

Authors and Year	Criteria 1	Criteria 2	Criteria 3	Criteria 4	Criteria 5	Criteria 6	Criteria 7	Criteria 8	Criteria 9	Criteria 10	Criteria 11	Total
Almeida et al., 2022 [30]	✓	✓	✓	✓	✓	✓	×	✓	✓	✓	✓	10
Amiri et al., 2023 [82]	✓	✓	✓	✓	✓	✓	×	✓	×	✓	✓	9
Arazi et al., 2019 [31]	✓	✓	✓	✓	✓	✓	×	✓	×	✓	✓	9

Ayoama et al., 2003 [32]	×	✓	✓	✓	✓	✓	×	✓	×	✓	✓	8
Azizi et al., 2011 [73]	✓	✓	✓	✓	✓	✓	×	✓	×	✓	✓	9
Becque et al., 2000 [33]	✓	×	✓	✓	✓	✓	×	✓	×	✓	✓	8
Bernat et al., 2019 [34]	✓	✓	✓	✓	✓	✓	×	✓	×	✓	✓	9
Bonilla et al., 2021 [35]	✓	✓	✓	✓	✓	×	×	✓	×	✓	✓	8
Brenner et al., 2000 [74]	×	✓	✓	✓	✓	✓	×	✓	×	✓	✓	8
Brooks et al., 2023 [75]	✓	✓	✓	✓	✓	✓	×	✓	×	✓	✓	9
Brose et al., 2003 [83]	✓	✓	✓	✓	✓	✓	×	✓	×	✓	✓	

												9
Camic et al., 2014 [36]	✓	✓	✓	✓	✓	✓	✗	✓	✗	✓	✓	9
Candow et al., 2015 [84]	✓	✓	✓	✓	✓	✓	✗	✓	✗	✓	✓	9
Candow et al., 2021 [37]	✓	✓	✓	✓	✓	✓	✓	✗	✓	✓	✓	10
Chami et al., 2019 [85]	✓	✓	✓	✓	✓	✓	✗	✓	✗	✓	✓	9
Chilibeck et al., 2015 [76]	✓	✓	✓	✓	✓	✓	✓	✗	✓	✓	✓	10
Chilibeck et al., 2023 [29]	✓	✓	✓	✓	✓	✓	✓	✓	✓	✓	✓	11
Chrusch et al., 2001 [38]	✓	✓	✓	✓	✓	✓	✗	✓	✗	✓	✓	9

Claudino et al., 2014 [39]	✓	✓	✓	✓	✓	✓	✕	✕	✕	✓	✓	8
Del Favero et al., 2012 [40]	✓	✓	✓	✓	✓	✓	✕	✓	✓	✓	✓	10
Earnest et al., 1995 [28]	✓	✓	✓	✓	✓	✓	✕	✕	✕	✓	✓	8
Eghbali et al., 2024 [41]	✓	✓	✓	✓	✓	✓	✕	✓	✕	✓	✓	9
Ferguson et al., 2006 [77]	✓	✓	✓	✓	✓	✓	✕	✓	✕	✓	✓	9
Forbes et al., 2017 [78]	✓	✓	✓	✓	✓	✓	✕	✓	✕	✓	✓	9
Furtado et al., 2024 [72]	✓	✓	✓	✓	✓	✓	✕	✓	✕	✓	✓	9
Gotshalk et al., 2002 [42]	✓	✓	✓	✓	✓	✓	✕	✓	✕	✓	✓	9

Green et al., 2001 [16]	✓	✓	✓	✓	✓	✓	×	✓	×	✓	✓	9
Gualano et al., 2014 [6]	✓	✓	✓	✓	✓	✓	×	×	×	✓	✓	8
Haff et al., 2000 [18]	×	✓	✓	✓	✓	✓	×	✓	×	✓	✓	8
Havenetidis et al., 2003 [43]	×	✓	✓	✓	✓	✓	×	✓	×	✓	✓	8
Herda et al., 2009 [44]	✓	✓	✓	✓	✓	✓	×	✓	×	✓	✓	9
Izquierdo et al., 2002 [45]	×	✓	✓	✓	✓	✓	×	✓	×	✓	✓	8
Javierre et al., 2004 [46]	✓	✓	✓	✓	✓	✓	×	✓	×	✓	✓	9
Johannsmeyer et al., 2016 [86]	✓	✓	✓	✓	✓	✓	✓	✓	×	✓	✓	10

Kaviani et al., 2019 [47]	✓	✓	✓	✓	✓	✓	✗	✓	✗	✓	✓	9
Kelly et al., 1998 [48]	✓	✓	✓	✓	✓	✓	✗	✓	✓	✓	✓	9
Kirksey et al., 1999 [87]	✗	✓	✓	✓	✓	✓	✗	✓	✗	✓	✓	8
Kresta et al., 2014 [79]	✓	✓	✓	✓	✓	✓	✗	✓	✗	✓	✓	9
Larson-meyer et al., 2000 [80]	✓	✓	✓	✓	✓	✓	✗	✓	✗	✓	✓	9
Law et al., 2009 [49]	✓	✓	✓	✓	✓	✓	✗	✓	✗	✓	✓	9
Mabrey et al., 2024 [50]	✓	✓	✓	✓	✓	✓	✗	✓	✗	✓	✓	9
Mills et al., 2020 [88]	✓	✓	✓	✓	✓	✓	✗	✓	✗	✓	✓	9

Mujika et al., 2000 [51]	✓	✓	✓	✓	✓	✓	✕	✓	✕	✓	✓	9
Noonan et al., 1998 [52]	✓	✓	✓	✓	✓	✓	✕	✓	✕	✓	✓	9
Okudan et al., 2014 [53]	✓	✓	✓	✓	✓	✓	✕	✓	✕	✓	✓	9
Pakulak et al., 2022 [89]	✓	✓	✓	✓	✓	✓	✕	✕	✕	✓	✓	8
Pearson et al., 1999 [54]	✕	✓	✓	✓	✓	✓	✕	✓	✕	✓	✓	8
Peeters et al., 1999 [55]	✓	✓	✓	✓	✓	✓	✕	✓	✕	✓	✓	9
Pinto et al., 2016 [90]	✓	✓	✓	✓	✓	✓	✓	✓	✕	✓	✓	10
Samadi et al., 2022 [56]	✓	✓	✓	✓	✓	✓	✕	✓	✕	✓	✓	9

Selsby et al., 2004 [57]	✓	✓	✓	✓	✓	✓	×	✓	×	✓	✓	9
Stone et al., 1999 [58]	✓	✓	✓	✓	✓	✓	×	✓	×	✓	✓	9
Stout et al., 1999 [59]	✓	✓	✓	✓	✓	✓	×	✓	×	✓	✓	9
Stout et al., 2007 [91]	✓	✓	✓	✓	✓	✓	×	✓	×	✓	✓	9
Syrotuik et al., 2000 [60]	✓	✓	✓	✓	✓	✓	×	✓	×	✓	✓	9
Syrotuik et al., 2001 [92]	✓	✓	✓	✓	✓	✓	×	✓	×	✓	✓	9
Tayebi et al., 2020 [61]	✓	✓	✓	✓	✓	✓	×	✓	×	✓	✓	9
Tayebi et al., 2021 [62]	✓	✓	✓	✓	✓	✓	×	✓	×	✓	✓	9

Theodorou et al., 2017 [63]	×	✓	✓	✓	✓	×	×	✓	×	✓	✓	7
Trexler et al., 2016 [64]	✓	✓	✓	✓	✓	✓	✓	✓	×	✓	✓	10
Vilar Neto et al., 2018 [65]	✓	✓	✓	✓	✓	✓	×	✓	×	✓	✓	9
Volek et al., 1999 [66]	✓	✓	✓	✓	✓	✓	×	✓	×	✓	✓	9
Volek et al., 2004 [67]	✓	✓	✓	✓	✓	✓	×	✓	×	✓	✓	9
Wang et al., 2016 [69]	✓	✓	✓	✓	✓	✓	×	✓	×	✓	✓	9
Wang et al., 2018 [68]	✓	✓	✓	✓	✓	✓	×	✓	×	✓	✓	9
Wilder et al., 2002 [70]	✓	✓	✓	✓	✓	×	×	✓	×	✓	✓	8

Williams et al., 2014 [71]	✓	×	✓	✓	✓	✓	×	✓	×	✓	✓	8
Zahabi et al., 2024 [81]	✓	✓	✓	✓	×	×	×	✓	×	✓	✓	7
Zuniga et al., 2012 [19]	×	✓	✓	✓	✓	✓	×	✓	×	✓	✓	8
